# Effect of the degree of blockiness on the glycosylation and emulsification of citrus pectin–whey protein isolate conjugates

**DOI:** 10.3389/fnut.2026.1929658

**Published:** 2026-08-26

**Authors:** Pengwei Huang, Guiqi Yuan, Xiaoyin Zhong, Jing Li, Yanbin Chen, Yingzhi Jiang

**Affiliations:** 1 Department of Chemical Engineering, Jieyang Polytechnic, Jieyang, China; 2 Department of Chemistry, Jinan University, Guangzhou, China; 3 South China Normal University, Guangzhou, China

**Keywords:** citrus pectin, degree of blockiness, emulsification, glycosylation, whey protein isolate

## Abstract

**Background:**

Maillard reaction-derived pectin–protein conjugates are common functional food emulsifiers. As a key structural parameter of citrus pectin (CP), the blockiness degree (DB) independently regulates the Maillard reaction between CP and whey protein isolate (WPI) and the emulsifying properties of their conjugates, which remains poorly understood. This study aimed to clarify such regulatory mechanisms to support the targeted development of high-performance pectin–protein emulsifiers.

**Method:**

CP samples with similar methoxylation degrees (34.2–36.7%) and varied DB (27.4–88.3%) were prepared via sequential enzymatic de-esterification with plant and fungal pectin methylesterases. Dry heating was subsequently applied to trigger Maillard conjugation between modified CP and WPI, and the emulsion test was performed.

**Result:**

DB significantly accelerated the glycation reaction, raising the glycation degree of CP–WPI conjugates from 48.2% to 66.7%. This improvement stemmed from increased local charge density and better accessibility of pectin neutral sugar side chains to WPI during dehydration. At equivalent glycation levels, high-DB conjugates exhibited lower surface hydrophobicity, thicker hydrated interfacial layers and stronger steric-electrostatic stabilization, with only slightly impaired interfacial adsorption. All conjugates showed better interfacial activity and emulsifying performance than native WPI.

**Conclusion:**

CP DB is a decisive factor governing the CP–WPI Maillard reaction efficiency and conjugate emulsifying functionality. Modulating pectin DB is a reliable strategy to fabricate pectin–protein emulsifiers with tunable functions, providing practical guidance for the preparation of high-efficiency food emulsifiers.

## 1 Introduction

Oil-in-water emulsions are widely used in food systems for delivering hydrophobic nutrients, flavors, and bioactive compounds. Whey protein isolate (WPI) is an effective natural emulsifier because of its amphiphilic structure and rapid adsorption at the oil–water interface (1). However, emulsions stabilized by WPI are unstable at the isoelectric point and high ionic strength due to the reduced electrostatic repulsion and protein aggregation (2).

The strategy of producing protein–polysaccharide conjugates via Maillard reaction has gained considerable attention as a food-compatible modification technique to improve the emulsification performance of protein (3–5). The covalent linkage formed between the *ε*-amino groups of lysine and reducing ends (or carbonyl groups) of polysaccharides provides strong steric stabilization and enhances the thickness and viscoelasticity of the interfacial film, thereby remarkably improving emulsion stability against coalescence and flocculation (4).

Pectin is a heteropolysaccharide mainly composed of *α*-1,4-linked D-galacturonic acid (GalA) in homogalacturonan regions that is interrupted by the insertion of 1, 2-linked L-rhamnose. The O-4 and O-3 positions of the rhamnose are linked with the neutral lateral chains, consisting mainly of D-galactose and L-arabinose (2). Previous studies have shown that WPI can be covalently conjugated with pectin through the Maillard reaction, thereby improving its emulsifying performance (2, 6). The formation and functionality of WPI–pectin conjugates are affected by pectin source, molecular weight, degree of methoxylation (DM), chemical composition, and reaction conditions (7–9). However, most current studies have focused on the overall amount of methoxy groups, while the effect of their distribution pattern along the pectin backbone remains unknown.

The degree of blockiness (DB) is a key structural parameter describing the distribution of methylesterified and non-methylesterified galacturonic acid residues in pectin (10). Unlike the DM, which only reflects the total proportion of methylesterified GalA residues and the amount of methoxy groups, DB determines the local charge density of pectin chains. Pectins with similar DM may therefore exhibit markedly different electrostatic characteristics and protein-binding behaviors. Previous studies have demonstrated that local charge density strongly influences the electrostatic complexation between pectin and *β*-lactoglobulin, the major protein component of WPI (11). Since WPI–pectin Maillard conjugation is closely related to the interaction between protein and polysaccharide molecules, DB may play an important role in regulating both the reaction process and the final interfacial properties of the conjugates.

Pectins with different DB can be prepared by enzymatic de-esterification using pectin methylesterase (PME) from different origins. PME from plants generally removes the methoxy groups in a blockwise manner, whereas PME from fungi works in a random manner (12, 13). This provides a feasible technique to obtain pectins with similar DM but different DB, allowing the independent role of methoxy group distribution to be investigated. It can be hypothesized that high-DB pectin, with stronger local negative charge density, may enhance the interaction between WPI and pectin and accelerate Maillard conjugation. However, the strengthened electrostatic repulsion after conjugation may also influence the interfacial adsorption and the thickness of the adsorbed layer.

Therefore, this work aimed to elucidate the effect of pectin DB on the Maillard reaction between citrus pectin (CP) and WPI and also the emulsifying properties of the resulting conjugates. CP and WPI were chosen for their high degree of commercialization, and they are highly representative. CP with varying DB were prepared enzymatically by using PME from orange peel and *fungus Aspergillus aculeatus*. The degree of glycation and structure changes of CP-WPI Maillard conjugates were further characterized to distinguish the effect of DB on the Maillard reaction. The emulsification performance, protein adsorption efficiency, and adsorbed layer thickness of the conjugates were investigated. This work provides new insight into how DB regulates WPI–pectin Maillard conjugation and offers a structural basis for designing pectin-protein emulsifiers with tunable interfacial properties.

## 2 Materials and methods

### 2.1 Materials

CP was the product of Herbstreith & Fox KG Pektin-Fabriken (Neuenbürg, Germany). WPI (protein content of 95.6%) was supplied by Davisco Foods International Inc. (Le Sueur, MN, USA). PME from orange peel (PME-O) and *fungus Aspergillus aculeatus* (PME-F) was purchased from Macklin Co., Ltd. (Shanghai, China) and AB Enzymes Co., Ltd. (Shanghai, China), respectively. Endo-polygalacturonanase from *Pectobacterium carotovorum* was provided by Megazyme Co., Ltd. (Wicklow, Ireland). Medium-chain triglyceride (MCT) was provided by Britz Networks Sdn. Bhd (Melaka, Malaysia). Galacturonic acid (GalA), digalacturonic acid (di-GalA), trigalacturonic acid (tri-GalA), pyrene, *m*-phenyl phenol, anilino-1naphthalenesulfonic acid (ANS), 2,4,6-trinitrobenzene sulfonic acid (TNBS), sodium dodecyl sulfate (SDS), NaN_3_, and other reagents were obtained from Sigma-Aldrich (Shanghai, China).

### 2.2 De-esterification of CP

The blockwise de-esterification of CP was carried out as reported (12). Pectin was dissolved in phosphate buffer (5 mM, pH 7.0) to reach a concentration of 2% w/v. De-esterification was triggered by adding PME-O (2 U/g pectin) into pectin solution (30 °C, 200 rpm).

The random de-esterification of CP was carried out as reported (13). Pectin was dissolved in citrate buffer (5 mM, pH 4.2) to reach a concentration of 2% w/v. De-esterification was triggered by adding PME-F (2 U/g pectin) into a pectin solution (30 °C, 200 rpm).

To fabricate CP with varying DB, two-step de-esterification was applied. CP was first hydrolyzed by PME-O as demonstrated above. At hydrolysis times of 15 min, 40 min, and 80 min, citric acid powder was added into the solution, and the pH was adjusted to pH 4.5 (the activity of PME-O at this pH was inhibited). Then, PME-F (2 U/g pectin) was added to the pectin solution at 30 °C under stirring (200 rpm), enabling the enzymatic reaction to continue.

After enzymatic modification, the sample solution was boiled (5 min), dialyzed (10 kDa cut off, 48 h against water), and lyophilized to recover the samples.

### 2.3 Characterization of CP

The GalA content was determined with the *m*-phenyl phenol method (14).

The degree of methoxylation (DM) was determined according to Levigne et al. (15) by high-performance liquid chromatography (HPLC). For the dried pectin sample, methanol was released from pectin by saponification with NaOH (0.4 M)-isopropanol (1:1). To quantify the methanol released for the enzymatic de-esterification, the solution was directly analyzed by a C_18_ column (SinoChrom ODS-BP, 250 × 4.6 mm, 5 μm; Elite Corp., Dalian, P. R. of China) and a refractive index detector (Waters 2,414), with 5 mM H_2_SO_4_ as a mobile phase at 0.6 mL/min (16).

The degree of blockiness (DB) was determined by high-performance anion exchange chromatography as reported by Zhang et al. (13). Briefly, mono/di/tri-GalA was released from pectin by the hydrolysis of Endo-polygalacturonanase, then analyzed by CarboPac PA200 column (4 × 250 mm) with NaOH (150 mM) and CH_3_COONa (800 mM) as mobile phase (flow rate of 1 mL/min). DB was calculated as follows (Equation 1):

DB(%)=(M+D+T)/Free GalA×100 (1)

where M, D, T, and Free GalA (mol) were the molar content of mono-GalA, di-GalA, tri-GalA, and non-esterified GalA in the pectin sample, respectively.

The content of neutral sugar (NS) was determined by high-performance anion exchange chromatography (HPAEC) with a pulsed amperometric detector (PAD) (17, 18). A 20 mg sample was enzymatically hydrolyzed using 2 mL of Viscozyme L (2 U) (Novozymes Company, Tianjin, China) at pH 5.0 for 24 h at 45 °C. The hydrolysate was then mixed with 2 mL of 4 M trifluoroacetic acid and incubated at 110 °C for 2 h. Samples were injected into a guard CarboPac PA1 column (4 × 50 mm) followed by a CarboPac PA1 column (4 × 250 mm) using 1 mL/min of 14 mM NaOH as an eluent at 35 °C. Rhamnose, arabinose, galactose, glucose, and xylose were used as standards for constructing the calibration curve, and the NS content was calculated as the sum of these five neutral sugars.

The molecular weight (*M*_w_) was measured by size exclusion chromatography coupled with a multi-angle light scattering detector (SEC-MALLs) (DAWN HELEOS, Wyatt Corp., California, USA) (19). Pectin was dissolved in to mobile phase (100 mM NaNO_3_ with NaN_3_ as preservative) and analyzed by Ultrahydrogel 2000 (300 mm × 7.5 mm) and Ultrahydrogel 1,000 Column (300 mm × 7.5 mm) (Waters Corp., USA) at 0.6 mL/min. The dn/dc was 0.135 mL/g for calculation (20).

The *ζ*-potential was measured by Zetasizer (Nano ZS, Malvern Ltd., UK) with a pectin concentration of 1 mg/mL (21).

Shear viscosity of pectin was recorded by a Discovery HR-2 rotational rheometer (TA Instruments Ltd., US) with a 40 mm diameter parallel plate geometry (1 mm gap) at a shear rate of 10 s^−1^ (22, 23).

### 2.4 Conjugation between CP and WPI

A dry-state Maillard reaction procedure was carried out to prepare the CP-WPI conjugates (24). CP and WPI with a weight ratio of 2:1 were dissolved into distill water to reach 30 mg/mL, and the pH was adjusted to 7.0. The mixture was lyophilized and heated at 70 °C with controlled humidity 79% (sealed in a desiccator containing saturated potassium bromide solution) for different times (0–72 h).

### 2.5 Characterization of CP-WPI conjugates

The degree of glycation (DG) was measured by a TNBS method (8).

The surface hydrophobicity (*H*_0_) was determined as reported (24, 25) by using ANS as a fluorescence probe. Emission fluorescence intensity at 480 nm, excited at 360 nm, was recorded and plotted against protein concentration, in which the initial slope was used as *H*_0_.

The secondary structure of the protein was accessed by circular dichroism spectrum as described by Luo et al. (2). The concentration of protein was set at 0.2 mg/mL in phosphate buffer (pH 7.4, 10 mM) and analyzed for the wavelength range 190–250 nm. The data of the secondary structure were calculated by the Circular Dichroism Neural Network (CDNN) program (version 2.1.0.223).

### 2.6 Interfacial tension and emulsion test

Interfacial tension measurement was carried out via the pendant-drop method using a tensiometer (OCA-20, DataPhysics, Germany). Protein concentrations of the tested samples were set at 5 and 15 mg/mL for WPI and CP-WPI conjugates in phosphate buffer (pH 7.0, 10 mM). A pendant drop (15 μL) of sample solution was formed by a syringe needle and immersed in MCT. The shape of the pendant drop was recorded by a CCD camera and the interfacial tension was calculated by SCA 20 software according to the Young-Laplace equation (8).

The emulsifying activity index (EAI) and emulsion stability index (ESI) were determined with a turbidimetric method (2). Emulsifier solution (20 mL, with protein solution of 5 mg/mL) was directly mixed with MCT (5 mL) and then homogenized (22,000 rpm, 1 min). Then, the fresh emulsion (50 μL) from the bottom was diluted with SDS (0.1% wt, 5 mL), and the absorbance at 500 nm was recorded as *A*_0_. After 30 min, the absorbance at 500 nm was recorded as *A*_30_. EAI and ESI were calculated as follows (Equations 2,3):

$$\mathrm{EAI}\;(m^{2}/g)=(2\times 2.303\times A_{0}\times N)/(\rho \times \Phi \times (1-\theta )\times 10,000)$$ (2)

$$\mathrm{ESI}\;(\min)=A_{0}/(A_{0}-A_{30})\times 30$$ (3)

Where *N* is the coefficient of dilution (*N* = 101), *ρ* is the emulsifier concentration (g/mL), Φ is the optical range of the measurement (cm), and *θ* is the MCT fraction (*θ* = 0.20).

The adsorbed protein was determined by the ultracentrifugation technique (26). Emulsifier with protein concentration of 0.5% wt was dispersed in phosphate buffer (pH 7.0, 10 mM). The mixture of 85 g emulsifier solution and 15 g MCT was first pre-homogenized at 20,000 rpm for 2 min and then passed through a high-pressure homogenizer for two cycles to obtain a fine emulsion (50 MPa). The fresh emulsion was centrifuged (800,000 × g for 30 min) to completely separate the emulsion and serum phase using an Optima XE-100 (Beckman Coulter, Inc., USA). The lower aqueous phase was drawn with a syringe. The content of protein remaining in serum was determined by the Lowry method (24).

The adsorbed layer thickness was evaluated by using polystyrene (PS) beads (200 nm, DAE Scientific Co., Ltd., Tianjin, China) as a model system (27). Emulsifier solution (protein concentration of 0.88 mg/mL, 4.88 mL) was mixed with PS beads (0.12 mL) to achieve 50 mg / m^2^ PS beads. After 8 h, the particle size was determined by dynamic light scattering (Zetasizer Nano ZS, Malvern Ltd., Worcestershire, UK). The adsorbed layer thickness was calculated as the variation in particle size before and after adsorption.

### 2.7 Statistical analysis

Experiments were conducted in triplicate and analyzed by Origin software (OriginPro learning edition) with one-way analysis of variance (Scheffe’s test, *p*-value < 0.05).

## 3 Results and analysis

### 3.1 De-esterification of pectin

**Figure 1:**
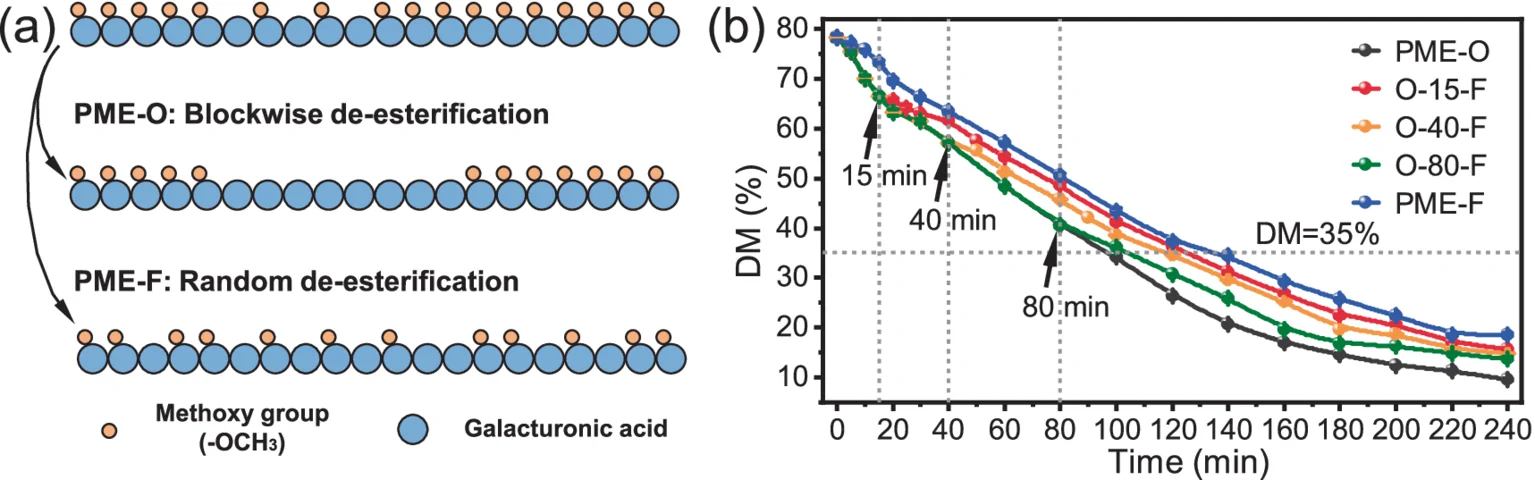
The schematic diagram of de-esterification by pectin methylesterase from orange peel (PME-O) and fungus (PME-F) **(a)**. Dependence of degree of methoxylation (DM) on the reaction time during the de-esterification **(b)**.

To investigate the influence of DB on the Maillard conjugation between CP and WPI, CP from different types of DB needed to be obtained, and the molecular structures (such as chemical composition and *M*_w_) should remain unchanged. This is in order for the investigation to be conducted by controlling variables. The enzymatic hydrolysis technique was selected in this work as shown in Figure 1a. PME from plants (PME-O) and fungi (PME-F) remove the methoxy groups from pectin via a blockwise and random manner, respectively (12, 28). The methanol released by enzymatic de-esterification was monitored by HPLC method, and the DM was calculated. Under the same enzyme addition (2 U/g pectin), PME-O showed higher enzymatic hydrolysis efficiency than PME-F as the DM decreased more rapidly (Figure 1b). Previously, Tanhatan-Nasseri et al. (29) proposed a two-step de-esterification technique, which sequentially used alkaline treatment and enzymatic hydrolysis to fabricate pectin with different DB. This strategy was adopted with modification. PME-O was first used for blockwise de-esterification. After observing evidence of reaction for 15 min (O-15-F), 40 min (O-40-F), and 80 min (O-80-F), the pH was instantly adjusted to 4.5 (the activity of PME-O was inhibited at this pH), and PME-F was added to trigger the random de-esterification. De-esterified CP obtained after different treatments with DM of ~35% (the commonly seen value of low methoxyl pectin) was selected for further characterization (9).

As expected, CP with different DB (27.4–88.3%) and similar DM (34.2–36.7%) were obtained as shown in Table 1. Due to the specificity of enzymatic hydrolysis, only the methoxy groups were PME target sites. There was no significant difference in GalA (74.9–76.5%) and NS (the sum of neutral monosaccharides, 16.4–17.8%) before and after de-esterification. The constant content of GalA of enzymatically de-esterified pectin agreed with the results reported by Daas et al. (30). The degradation was greatly inhibited as the *M*_w_ slightly decreased from 534.1 kg/mol to 511.8–518.4 kg/mol. Since GalA and NS are the component units that construct the main chain and side chain of pectin, the above results suggest that molecular integrity was retained after the enzymatic de-esterification (31). Similar protection of molecular structures by using PME for de-esterification was also reported by Ralet et al. (32), where the *M*_w_ of pectin was slightly decreased from ~120 kg/mol to ~110 kg/mol.

**Table 1:** Chemical composition of citrus pectin.^a^

Parameter	CP	C27	C38	C62	C75	C88
GalA (% w/w)	75.8 ± 1.7a	76.4 ± 2.1a	74.9 ± 1.6a	75.4 ± 2.2a	76.1 ± 1.8a	76.5 ± 1.9a
NS (% w/w)	17.8 ± 0.9a	16.4 ± 0.7a	17.1 ± 0.5a	17.3 ± 0.6a	16.7 ± 0.7a	16.9 ± 0.5a
DM (% mol)	81.3 ± 1.2d	35.5 ± 0.5b	34.2 ± 0.7a	35.9 ± 0.4b	36.7 ± 0.3c	35.8 ± 0.4b
DB (% mol)	10.6 ± 0.8a	27.4 ± 1.2b	38.3 ± 0.9c	62.1 ± 1.3d	74.6 ± 1.9e	88.3 ± 2.6f
*M*_w_ (kg/mol)	534.1 ± 7.5b	511.8 ± 5.8a	512.7 ± 7.6a	518.4 ± 4.6a	514.5 ± 9.4a	517.6 ± 3.7a
*ζ*-potential (mV)^b^	−28.7 ± 1.8a	−32.4 ± 1.3b	−35.2 ± 1.1c	−45.7 ± 2.0d	−49.6 ± 1.2e	−52.9 ± 1.4f
Viscosity (mPa·s) ^c^	125.2 ± 3.1f	85.7 ± 1.9e	81.5 ± 1.2d	74.2 ± 2.3c	66.9 ± 1.8b	63.4 ± 1.3a

a Data are means ± standard deviations (*n* = 3). Values in the same row followed by different letters are significantly different (*p* < 0.05).

b Pectin concentration of 1 mg/mL at pH 7.0.

c Viscosity was measured at a shearing rate of 10 s^−1^ and pH 7.0 with pectin concentration of 5 mg/mL (39). Citrus pectin (CP) with degrees of blockiness (DB) of 27.4, 38.3, 62.1, 74.6%, and 88.3 were named C27, C38, C62, C75, and C88, respectively.

### 3.2 The Maillard reaction between CP and WPI

#### 3.2.1 Degree of glycation (DG)

**Figure 2:**
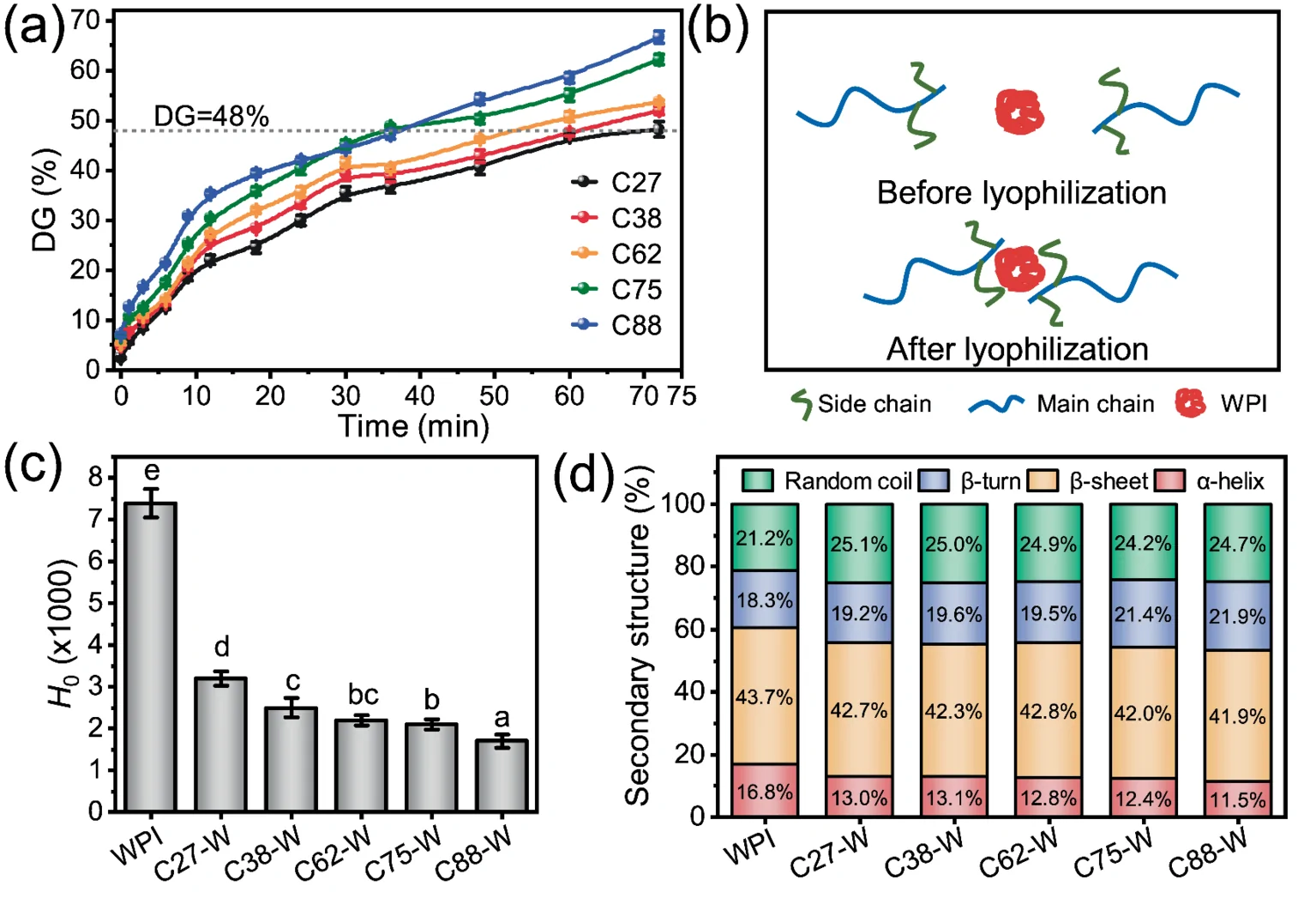
Dependence of degree of glycation (DG) on the reaction time during the Maillard reaction **(a)**. The schematic diagram of citrus pectin (CP) and whey protein isolate (WPI) interaction during lyophilization **(b)**. The surface hydrophobicity (*H0*) **(c)** and secondary structure **(d)** of WPI and CP-WPI conjugates.

The retrieval of CP with different DB (but other chemical composition was constant, as seen in Table 1) enabled investigation into the independent role of methoxy groups distribution on the Maillard reaction between CP and WPI. CP with DB of 27.4, 38.3%, 62.1%, 74.6%, and 88.3 were named as C27, C38, C62, C75, and C88, respectively. The Maillard reaction between CP and WPI was investigated by monitoring the degree of glycation (DG). The DG progressively increased over the duration of the reaction (72 h, Figure 2a), suggesting the free amino groups of WPI were consumed by the Maillard reaction with reducing ends (or carbonyl groups) of CP (8). The DG of CP-WPI conjugates increased from 48.2 to 66.7% as the DB increased from 27.4 to 88.3%, indicating the blockwise distribution of methoxy groups promoted conjugation between CP and WPI. By using enzymes to specifically hydrolyze the homogalacturonan and the arabinan/galactan, Guo et al. (8) and Lin et al. (24) found that the Maillard reaction between protein and polysaccharide was more likely to occur in the neutral sugars region as compared to the galacturonic acid region, as the neutral sugar side chains were rich in reducing ends. In the present work, CP and WPI were mixed at pH 7.0 and then freeze-dried for the Maillard reaction. Both the WPI and CP were observed to be negatively charged at pH 7.0, and the *ζ*-potential of CP with higher DB was increased from −32.4 mV (C27) to −52.7 mV (C88) (Table 1). The decreased *ζ*-potential of pectin with high DB was also reported by Lutz et al. (33) and Sperber et al. (11), and this was ascribed to the increase in local charge density at the main chains (homogalacturonan) of pectin due to the blockwise distribution of methoxy groups. The increase in local charge density strengthened the electrostatic repulsion between the homogalacturonan main chains of CP and WPI. Consequently, the main chains shifted farther away from WPI in solution, whereas the neutral sugar side chains reoriented toward WPI (Figure 2b). Although stronger electrostatic interactions increased the distance between CP and WPI prior to lyophilization, the removal of water during lyophilization enabled the neutral sugar side chains to approach WPI more closely, thus promoting the Maillard reaction during heating of the dried CP-WPI mixtures. The increased *ζ*-potential also indicated the weakening of intermolecular interactions between CP (34), which was reinforced by the viscosity measurement. The entanglement between pectin chains diminished as the viscosity decreased (from 85.7 to 63.4 mPa·s) with increased DB (Table 1). Similar results were also reported by Lutz et al. (33).

To make the subsequent characterization and emulsification performance analysis more reasonable, we selected the CP-WPI conjugates with similar DG (~48%) for the subsequent analysis: C27-W, 48.2%; C38-W, 47.5%; C62-W, 50.8%; C75-W, 48.7%; C88-W, 47.2%.

#### 3.2.2 Surface hydrophobicity (H0)

Surface hydrophobicity (*H*_0_) was used in this work as an indicator of protein conformational changes during the Maillard reaction (35). As compared to WPI, the conjugation of CP with different DB significantly reduced the *H*_0_ (Figure 2c), implying that some ANS-accessible hydrophobic sites on WPI were buried by robust polysaccharide chains of pectin. Similar decreases in *H*_0_ were observed in the conjugates of sugar beet pectin-WPI and soybean soluble polysaccharide-soy protein isolate (24, 35). The *H*_0_ value of the conjugates gradually decreased (from 3,226 to 1,695) with increasing DB, suggesting that the increased local charge density of CP further blocked the binding between ANS and the WPI. By the analysis of small-angle X-ray scattering, Alba et al. (36) found that the conformational flexibility of pectin increased with higher DM and acidic pH, which was attributed to the decreased charge density resulting from protonation of the galacturonic acid. Pectin chains with higher conformational flexibility were prone to entanglement, thereby forming a more compact conformation in aqueous solution. For CP-WPI conjugates, the higher DB CP with increased local charge density promoted the electrostatic repulsion of negatively charged pectin chains. Therefore, the polysaccharide chains covalently bound to the surface of WPI were extended, forming a thicker and more robust pectin layer to prevent ANS from binding to WPI.

#### 3.2.3 Secondary structure

The analysis of the secondary structure of the protein is shown in Figure 2d. It was found that unfolding and conformational rearrangement of WPI was driven by the Maillard reaction, as indicated by the decreased content of *α*-helix (from 16.8 to 11.5–13.0%) and *β*-sheet (from 43.7 to 41.9–42.7%) as well as the increase in *β*-turn (from 18.3 to 19.2–21.9%) and random coil (from 21.2 to 24.7–25.1%). The transformation of *α*-helix and *β*-sheet to *β*-turn and random coil WPI after the Maillard reaction suggested that the structure of WPI became unfolded and disordered. Similar structural transformation was also reported for the pectin-WPI conjugates by Luo et al. (2). Although the structural transformation could promote the exposure of hydrophobic sites of WPI and increase the *H*_0_, the remarkable decrease in *H*_0_ after conjugation (Figure 2c) confirmed that the conjugated CP blocked the binding between ANS and WPI (37).

### 3.3 Emulsification performance

#### 3.3.1 Interfacial tension

**Figure 3:**
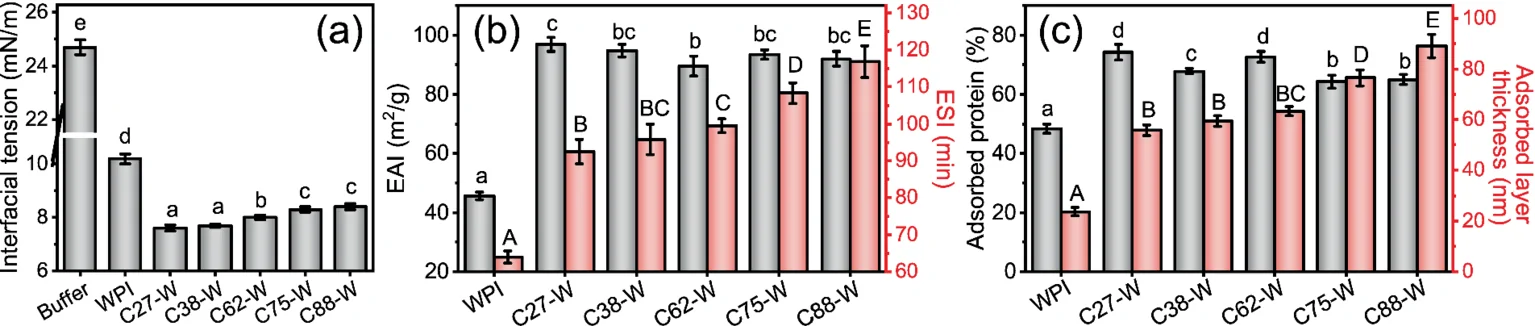
The interfacial tension **(a)**, emulsifying activity index (EAI), emulsion stability index (ESI) **(b)**, adsorbed protein, and adsorbed layer thickness **(c)** of whey protein isolates (WPI) and citrus pectin (CP)-WPI conjugates.

Prior to the emulsion test, the interfacial tension of WPI and CP-WPI conjugates was determined to evaluate the interfacial activity. As shown in Figure 3, all the WPI and CP-WPI conjugates showed superior interfacial activity due to their oil–water interfacial tension (7.6–10.2 mN/m) being significantly smaller than that of phosphate buffer (24.7 mN/m). The improved interfacial activity might be attributed to the unfolding of WPI by the conjugation with CP. Compared to WPI (10.2 mN/m), all the CP-WPI conjugates showed improved interfacial activity with significantly smaller interfacial tension (7.6–8.4 mN/m), which agreed with the reports of sugar beet pectin-WPI conjugates (8) and soybean soluble polysaccharide*−*soy protein isolate conjugates (24). The improved emulsifying activity of conjugates may be ascribed to the unfolding and disordered structure of WPI, which allowed it to make contact with the oil phase. Conjugates with higher DB exhibited an increasing trend toward interfacial tension (from 7.6 to 8.4 mN/m). Lin et al. (26) proposed that the accessibility of protein was vital to the interfacial adsorption of colloidal emulsifier. As indicated by the decreased *H_0_* (Figure 2c), the thicker and more robust pectin layer conjugated on the surface of WPI would hinder the exposure of protein to the oil–water interface, thus diminishing interfacial activity.

#### 3.3.2 Emulsifying properties and interfacial adsorption

The emulsifying properties of WPI and CP-WPI conjugates were systematically evaluated by EAI and ESI (Figure 3b). All the conjugates exhibited enhancement in both EAI and ESI, which could be explained by the balanced amphiphilicity and thicker adsorbed layer. First, the conjugation of hydrophilic pectin chains to the WPI decreases the *H*₀ (Figure 2c), leading to a more suitable hydrophilic–hydrophobic balance (38). This structural adjustment facilitates the transport of the conjugates from the continuous phase to the oil–water interface and favors their efficient anchoring at the interface, thus decreasing the interfacial tension (Figure 3a). Second, pectin is a highly hydrated and negatively charged polysaccharide of bulky molecular size and a branching structure. Once adsorbed onto the oil–water interface, the extended pectin chains generate a thick hydration layer around oil droplets, which enhances emulsion stability by providing steric protection together with electrostatic repulsion (35). As a result, the droplet–droplet contact is diminished, and emulsion destabilization caused by flocculation and coalescence is inhibited.

**Figure 4:**
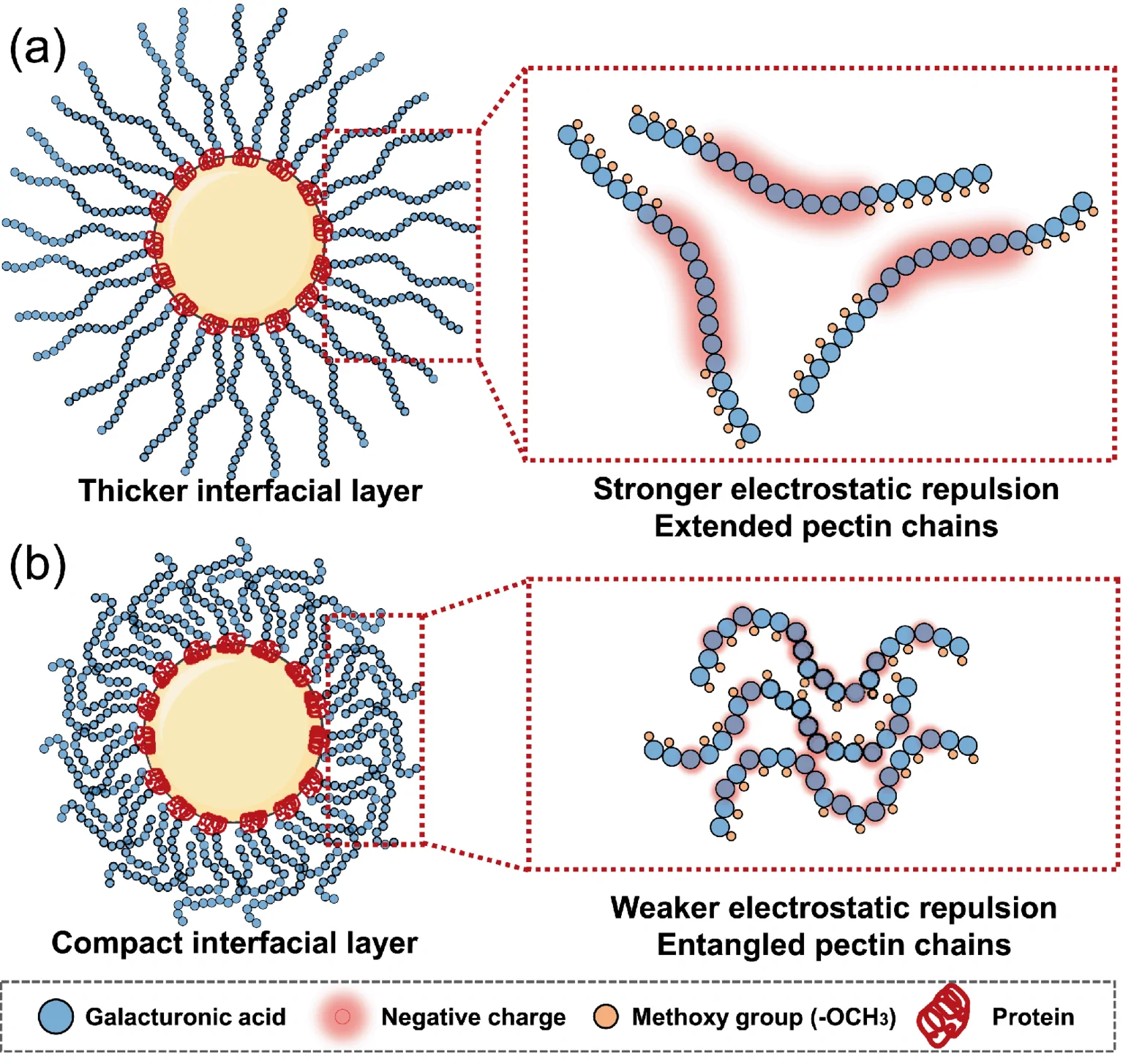
The schematic diagram of emulsions stabilized by the high **(a)** and low **(b)** degree of blockiness (DB) of citrus pectin (CP)-whey protein isolate (WPI) conjugates.

The EAI of CP-WPI conjugates showed a slightly decreasing trend (Figure 3b) as the local charge density was significantly increased with increasing DB (Table 1), which was probably ascribed to the decreased interfacial activity (Figure 3a). The inferior interfacial activity was reinforced by the interfacial adsorption test (Figure 3c). Maillard conjugation between CP and WPI significantly enhanced the protein adsorption at the oil–water interface, as adsorbed protein increased from 48.4% (WPI) to 64.3–74.4%. Similar adsorbed protein was also reported for soybean soluble polysaccharide-soy protein isolate conjugates (~75%) (24). However, increasing DB would slightly hinder the protein adsorption, which was attributed to the progressively enlarged adsorbed layer thickness (Figure 3c). The thicker adsorbed layer indicated that the pectin chains of CP-WPI conjugates protruded into the aqueous phase, which resulted from the stronger electrostatic repulsion between pectin chains with higher local charge density [evidenced by the result of lower *ζ*-potential of CP with high DB (Table 1; Figure 4a)]. The extended pectin chains (CP with high DB) attached to the WPI hinder the exposure of protein during homogenization, thus inhibiting the interfacial adsorption of protein and decreasing the EAI (26, 27). Although the electrostatic repulsion of CP with lower DB was weaker than that with higher DB, the pectin chains tended to entangle with enhanced intermolecular interaction (33). When adsorbed onto the oil–water interface, the entangled pectin chains of CP-WPI conjugates with lower DB formed a compact interfacial layer, but the thickness was thinner (Figure 4b). It has been widely reported that globular proteins will undergo structural unfolding and interfacial crosslinking to form the interfacial layer, but the thin adsorbed layer was sensitive to the external environment and thus unstable (1, 38). Regardless of the DB, the adsorbed layer became remarkably thicker after Maillard conjugation (Figure 3c), thus improving the emulsion stability (supported by Figure 3b). Our results indicated that the thicker interfacial layer formed due to stronger electrostatic repulsion improves the stability of the emulsion significantly compared to the compact but thinner interfacial layer.

## 4 Conclusion

This study demonstrated that the degree of blockiness (DB) is a key structural factor regulating the Maillard conjugation between citrus pectin and WPI and the emulsifying performance of the resulting conjugates. Pectins with similar DM but different DB were successfully prepared by enzymatic de-esterification while maintaining molecular integrity. Increasing DB enhanced local charge density and promoted glycation, which was ascribed to the improved accessibility of neutral sugar side chains to WPI during dehydration. At comparable glycation levels, higher-DB conjugates formed more extended and hydrated interfacial layers, which improved emulsion stability, although excessive DB slightly limited protein exposure and interfacial adsorption. Overall, DB was vital for designing pectin–protein emulsifiers with tunable Maillard reactivity and interfacial stabilization capacity.

## Data Availability

The original contributions presented in the study are included in the article/supplementary material, further inquiries can be directed to the corresponding author.
